# Arterial Elasticity, Strength, Fatigue, and Endurance in Older Women

**DOI:** 10.1155/2014/501754

**Published:** 2014-01-08

**Authors:** Gary R. Hunter, William H. Neumeier, C. Scott Bickel, John P. McCarthy, Gordon Fisher, Paula C. Chandler-Laney, Stephen P. Glasser

**Affiliations:** ^1^Department of Human Studies, University of Alabama at Birmingham, Birmingham, AL 35294, USA; ^2^Department of Nutrition Science, University of Alabama at Birmingham, Birmingham, AL 35294, USA; ^3^Department of Psychology, University of Alabama at Birmingham, Birmingham, AL 35294, USA; ^4^Department of Physical Therapy, University of Alabama at Birmingham, Birmingham, AL 35294, USA; ^5^Department of Human Movement Sciences, Carroll University, Waukesha, WI 53186, USA; ^6^Department of Preventive Medicine, University of Alabama at Birmingham, Birmingham, AL 35294, USA

## Abstract

Arterial health may influence muscle function in older adults. Study purpose was to determine whether arterial elasticity is related to strength, central and peripheral fatigue, fatigue at rest, and treadmill endurance. Subjects were 91 healthy women aged >60. Treadmill endurance and maximal oxygen uptake (VO_2_ max) were measured. Peripheral and central fatigue for the knee extensors were evaluated using two isometric fatigue tests (one voluntary and one adding electrical stimulation). Arterial elasticity was determined using radial artery pulse wave analysis. Linear multiple regression was used in statistical analysis. Large artery elasticity was associated with central fatigue (*P* < 0.01) and treadmill endurance (*P* < 0.02) after adjusting for VO_2_ max and knee extension strength. Subjective fatigue at rest was related to large artery elasticity after adjusting for ethnic origin (<0.02). Strength was significantly related to small artery elasticity after adjusting for ethnic origin, leg lean tissue, age, and blood pressure. Arterial elasticity is independently related to strength and fatigue in older women, especially in the central nervous system where arterial elasticity is independently related to perceptions of fatigue at rest and central fatigue. These results suggest that arterial health may be involved with the ability of the central nervous system to activate muscle in older women.

## 1. Introduction

A large proportion of older adults commonly report fatigue [1]. In addition, older adults who complain of fatigue have poor functional status compared with those who report less fatigue [2]. Finally, central fatigue (a brain mediated decreased ability to activate muscle neurons) is a poorly understood phenomena that contributes to fatigue in both young and old. For example, central fatigue is not increased predictably with elevated brain temperature or decreased glucose levels [3]. Although it might be considered a safety mechanism, exercise training reduces central fatigue during fatiguing intense exercise.

The brain receives 15% of cardiac output and 20% of oxygen uptake at rest [4]. Cerebral blood flow decreases after the age of 30 years [5] with adults aged 71 having cerebral blood flows 20–30% less than those aged 27. Endothelial dysfunction is one of the earliest indicators of structural and functional changes in the aging vascular system and arterial elasticity seems to be related to endothelial dysfunction [6]. Reduced elasticity may alter steady state blood flow to the brain which may affect the delivery of sufficient nutrients, impacting brain function and increasing feelings of fatigue.

To date, there is limited information available concerning the relationship between arterial elasticity, fatigue, and muscle function. Recently, Fahs et al. [7] reported that muscular strength was inversely related to aortic stiffness independent of aerobic fitness, whereas Heffernan et al. [8] found a significant relationship between vascular function and muscular power but not strength. However, it appears neither study adjusted for the potential confounder muscle size. Some [9] but not all [10] studies find that aerobic exercise training improves arterial stiffness, while older aerobic athletes have lower pulse wave velocity than sedentary peers [9]. In addition, reduced gate performance [11] and poor physical function [12] are related to arterial stiffness in healthy older adults. Increased aortic stiffness is associated with reduced exercise capacity in patients with hypertrophic cardiomyopathy [13] while increased pulse wave velocity is associated with reduced walking distance in patients with peripheral arterial disease and arterial stiffness is associated with reduced walking ability in end stage renal disease [14]. Few of these studies have determined whether the relationships are confounded by variation in aerobic fitness, strength, body composition, or age.

Understanding muscle function and fatigue is particularly important in older women since they have reduced muscle function compared to older men and are therefore vulnerable at earlier ages for loss in muscular function [15]. In addition, fatigue increases with age and increases more rapidly in older women than older men [2]. The purpose of this study is to determine whether arterial elasticity is related to (1) strength; (2) central and peripheral fatigue during a fatiguing maximal isometric contraction protocol; (3) subjective measures of fatigue; and (4) treadmill endurance independent of potential confounders such as muscle size, % fat, aerobic fitness, and age. We hypothesize that arterial elasticity will be negatively related to fatigue and positively related to strength and exercise endurance in older women after adjusting for appropriate confounders.

## 2. Materials and Methods

### 2.1. Experimental Approach to the Problem

In order to test these hypotheses, healthy and sedentary older women were recruited to participate in exercise testing and measures of aerobic capacity, body composition, and fatigue. Central and subjective fatigue measures were chosen to obtain objective and subjective reports. Variables such as strength, age, percent body fat, and aerobic fitness were considered potential confounders in exploring the relationship between arterial elasticity and fatigue.

### 2.2. Subjects

Ninety-one healthy, sedentary (self-reporting no exercise training to the Program Coordinator at initial screening), postmenopausal African American (*n* = 15) and American women of European decent (European American, *n* = 76) who are 60 or older volunteered to participate in this study designed to determine factors that may influence fatigue in older women. Eighteen of the 91 subjects reported taking hormone replacements.

Exclusion criteria included heart disease, abnormal EKG (both at rest and during exercise), smoking, diabetes mellitus, participation in regular physical activity (more than once/week), or medications that affected energy expenditure, insulin levels, thyroid status, or heart rate. Methods and procedures were approved by the appropriate institutional review board, and all subjects gave verbal and written informed consent.

### 2.3. Procedures

#### 2.3.1. Resting Systolic Blood Pressure (SBP)

Resting blood pressure was taken by automatic auscultation (Omron Blood Pressure Monitor, model HEM-780; Omron Healthcare, Inc 1200 Lakeside Dr. Bannockburn, IL) while lying in a supine position. Readings were taken after 12 hours of fast between 7 : 00 and 8 : 00 AM.

#### 2.3.2. Maximal Exercise Testing

It was physician-supervised and conducted using the modified Balke treadmill test protocol. A metabolic cart, calibrated prior to testing (Vmax Spectra29, SonsorMedics, Inc, Yorba Linda, CA), was used to evaluate ventilatory expired gases. Monitoring consisted of 12-lead electrocardiogram and BP measurements taken every two minutes (Omron Blood Pressure Monitor, model HEM-780; Omron Healthcare, Inc 1200 Lakeside Dr. Bannockburn, IL). The testing was commenced with treadmill walking at two mph for two minutes. Treadmill grade was increased by 3.5% every 2 minutes until minute 12 at which time grade was decreased to 12% and speed was increased to 3 mph. The grade then increased by 2.5% each minute until exhaustion. Blood pressure, heart rate, and oxygen uptake were recorded during the last 20 seconds of each level. Participants were encouraged to exercise to fatigue. Termination criteria for testing followed American Heart Association/American College of Cardiology guidelines [16]. Maximum oxygen uptake (VO_2_ max in mL·kg^−1^·min^−1^), maximum respiratory exchange ratio (RER), and maximum heart rate were defined as the highest 20-second averaged value. Criteria for obtaining maximum oxygen uptake were heart rate within 10 beats of age predicted maximum, plateauing of oxygen uptake, and RER of 1.1 or larger. Only those subjects achieving at least two of these criteria were included in the analysis of VO_2_ max and treadmill endurance (77 subjects). Treadmill endurance was considered the time spent on the treadmill until voluntarily stopping the test.

#### 2.3.3. Torque Measures

Torque measurements were performed in the knee extensor muscle group using a custom-built chair with the hip and knee secured at approximately 90 degrees of flexion. The leg was secured to a rigid lever arm in an effort to ensure that the knee extensors would perform only isometric contractions. A load cell was attached via a steel rod to the rigid lever arm on one end and an immovable support on the other. The line of pull on the load cell was perpendicular to the rigid lever arm. Thus, the torque produced about the knee was calculated as the product of the load cell force and the length of the lever arm between axis of rotation and the load cell attachment. Prior to data collection, subjects were allowed to perform several warm-up contractions and were familiarized with performing maximum isometric contractions. Maximal voluntary isometric contraction (MVIC) was defined as the peak isometric torque achieved during 3 consecutive maximal efforts (~5 second contraction separated by 120 seconds of rest). Contraction intensity for subsequent neuromuscular electrical stimulation (NMES) testing was calculated relative to each subjects' MVIC.

#### 2.3.4. Central Activation Ratio (CAR)

Bipolar self-adhesive neuromuscular stimulation electrodes (7 × 10 cm) were placed over the distal-medial and proximal-lateral portion of the quadriceps muscle group, as reported previously [17]. Stimulation pulses were delivered using a Grass S88 stimulator with a Grass Model SIU8T stimulus isolation unit (Grass Technologies, West Warwick, RI). Stimulation pulses were 450 *μ*s in duration and delivered at 100 Hz for 100 ms (10 pulses); voltage was set at 125 V. Participants were familiarized with maximum voluntary isometric contraction (MVIC). During MVIC testing, the burst of stimulation was applied. CAR was calculated: MVIC torque/(MVIC torque + Stimulation). The 30-repetition fatigue test (CAR30) was considered central fatigue. CAR was measured on only 40 of the subjects because data was collected on only the last 50 subjects entered in the study and because of inability of 10 subjects to complete protocol.

#### 2.3.5. Fatigue Tests

Fatigue tests consisted of voluntary and NMES 30-repetition MVIC protocols (40 subjects). The voluntary fatigue protocol consisted of a series of MVICs, where each contraction was 5 seconds in duration with 5 seconds rest between contractions for 5 minutes (30 total contractions). CAR testing was administered and calculated during the 1st and 30th contraction of the voluntary fatigue test. A standard clinical electrical stimulator (Rich-Mar, Chattanooga, TN) was utilized for the NMES-induced fatigue protocol (50 Hz, 450 *μ*sec biphasic pulses) and also consisted of contractions that were 5 seconds on and 5 seconds off for 5 minutes (30 total contractions).

#### 2.3.6. Body Composition

Total fat, lean tissue, and leg lean tissue were measured using dual-energy X-ray absorptiometry (Prodigy; Lunar Radiation, Madison, WI). The scans were analyzed with the use of the GE Lunar enCORE software standard analysis module. Total body and right and left leg analyses were performed by adjusting *Total Body Cut* lines using the software's *ROI* (Region Of Interest) tool. Leg ROI's required adjusting the following Total Body Cuts: the *Pelvis Top* cut was placed immediately superior to the iliac crests; the *Left and Right Pelvis* cuts were adjusted tangential to the ischium and passing through the femoral necks without touching the pelvis; the *Left and Right Leg* cuts were adjusted to separate the hands and forearms from the legs; the *Center Leg* cut was adjusted to separate the right and left leg.

#### 2.3.7. Arterial Elasticity

Noninvasive radial artery pulse wave analysis was used for measurement of arterial elasticity (which in turn is related to endothelial function) [6]. Pulse wave analysis was performed in duplicate, and average values were reported. The radial artery waveform was obtained with a sensor positioned over the brachial artery and calibrated using an oscillometric method on the opposite arm. Thirty seconds of analog waveforms were digitized at 200 samples/sec, and a beat marking algorithm determined the beginning of systole, peak systole, onset of diastole, and end of diastole for all beats in the 30 sec measurement period. An average beat determination was constructed, and a parameter estimating algorithm (Hypertension Diagnostics, Eagan, MN) was applied to define a third-order equation that replicated the diastolic decay in the waveform. Small artery and large artery elasticity were then determined based on the modified Windkessel model [18]. The method used was the predecessor of the CR 2000 and DO 2020 instruments (Hypertension Diagnostics), which incorporates a new transducer and similar software with a revised analytical algorithm.

#### 2.3.8. Self-Reported Fatigue

Perceptions of fatigue were assessed with relevant items from the Beck Depression Inventory-ll (BDI) [19]. Participants completed the BDI at home, during the week prior to strength and physical function testing. Items 15 (loss of energy) and 20 (tiredness or fatigue) were the only two that were included in these analyses, with higher scores indicating greater loss of energy or fatigue. Self-reported fatigue was measured on 74 subjects due to incomplete/incorrect questionnaires.

### 2.4. Statistical Analyses

Means and standard deviations were calculated for all variables of interest. Pearson product correlations were determined for all contrasts of interest. Reported hormone replacement therapy was not related to any fatigue, endurance, or function measure so it was not included in the results. In order to determine the independent relationship between arterial elasticity variables and performance measures stepwise linear multiple regression was used to model central fatigue, treadmill endurance, fatigue/tiredness, and knee extension strength after adjusting for potential confounders. Potential confounders considered were VO_2_ max, knee extension strength, age, and percent body fat for central fatigue, treadmill endurance, and fatigue/tiredness. Potential confounders for strength were lean tissue and age. Only those potential confounders that had significant Pearson product correlations with the performance measures of interest were included in the multiple regression models. Multicollinearity was within acceptable limits for all models with variance inflation factor (1/1 − *R*_*i*_^2^) less than 1.5 for all variables in all models. The Central Activation Ratio (CAR) was not added to the study until the final two years, hence the reduced sample size for analyses including this variable (*n* = 40). Because of missing data for several variables, due to equipment malfunction and sickness, correlations and regression models have varying observations as indicated in the tables. IBM SPSS Statistics version 20 was used in the analyses. An alpha level of *P* < 0.05 was the criteria for significance.

## 3. Results

Descriptive characteristics are contained in Table 1.  Table 2 is a correlation table showing simple unadjusted associations between potential fatigue mediators and the various measures of performance/fatigue. Age was negatively related to treadmill endurance and both large and small artery elasticity. Percent fat was negatively related to treadmill endurance, loss of energy, and large artery elasticity. Leg lean tissue was related to maximum voluntary contraction, maximum voluntary contraction plus electrical stimulation, and small artery elasticity. Maximum oxygen uptake was positively related to treadmill endurance and large artery elasticity. Maximum voluntary contraction and maximum voluntary contraction plus electrical stimulation were highly related to each other, as well as both large and small artery elasticity. Loss of energy was significantly related to fatigue/tiredness and negatively related to large artery elasticity, while fatigue/tiredness was negatively related to both large and small artery elasticity. Systolic blood pressure was related only to small artery elasticity.

Based upon intercorrelations with fatigue measures and known potential confounding relationships between performance/fatigue and arterial elasticity, regression equations were developed that were designed to observe the independent relationship between arterial elasticity and the various fatigue measures.  Table 3 shows that large artery elasticity remains significantly related to central fatigue after adjusting for knee extension strength (maximal voluntary contraction) and VO_2_ max (partial *R* = − 0.46, *P* < 0.01). Model 1 in Table 4 shows that large artery elasticity is independently related to treadmill endurance independent of VO_2_ max, knee extension strength, and age (partial *R* = 0.42, *P* < 0.02). Because of limitations in sample size a potential confounder % fat was not included in Model 1. Therefore a second model was developed for treadmill endurance. After adjusting for VO_2_ max, knee extension strength, and % fat, large artery elasticity remained independently related to treadmill endurance. Subjective feelings of fatigue/tiredness are modeled in Table 5. Large artery elasticity is negatively related to fatigue/tiredness independent of small artery elasticity (partial = − 0.29,  *P* < 0.03) while small artery elasticity approached a significant negative relationship with fatigue/tiredness after adjusting for large artery elasticity (partial *R* = − 0.22,  *P* < 0.09). Finally small artery elasticity was related to knee extension strength (maximum voluntary contraction) after adjusting for leg lean tissue and age (Table 6, partial = 0.52,  *P* < 0.01).

## 4. Discussion

As we hypothesized, arterial elasticity was independently related to fatigue. This is particularly apparent when observing variables that are controlled by the central nervous system. Not only there was a relationship between large artery elasticity and fatigue/tiredness and lack of energy, but also central activation both during one maximal contraction and during a fatiguing maximal contraction protocol of 30 repetitions was related to arterial elasticity. In addition, similar to Fahs et al. [7] where arterial stiffness was inversely related to strength, we found that strength was positively related to arterial elasticity. This relationship occurred during activation of muscle voluntarily and voluntarily with electrical stimulation superimposed. It is tempting to hypothesize that arterial elasticity may positively affect both central (brain) and peripheral (muscle) function; however relationships do not prove cause and effect and could result from confounding of some other variable. However, we considered known potential confounders, such as age, strength (for fatigue measures), aerobic fitness, and leg lean tissue (surrogate of muscle), and still observed similar relationships. Thus the results of this study are supportive of the concept that arterial elasticity may influence central nervous system drive as well as muscle function.

Elasticity of both large and small arteries was related to leg strength (Table 2). This was the case whether strength was evaluated using just voluntary contractions or voluntary contractions plus electrical stimulation suggesting that the relationship was largely due to function of skeletal muscle. Small artery elasticity seemed to be more strongly related to strength than large artery elasticity. In addition, small artery elasticity continued to be related after adjusting for leg lean tissue (a surrogate of leg muscle [20]) and age. We know of no studies that have shown that arterial elasticity is related to muscular strength adjusted for volume of muscle, indicating that muscle quality/specific strength was enhanced in the individuals who possessed more arterial elasticity.

On the other hand large artery elasticity but not small artery elasticity was related to both reduced perceived fatigue/tiredness while at rest as well as central fatigue during maximal isometric contractions suggesting that brain function may be influenced by arterial elasticity. It is possible that arterial elasticity may be just a marker of overall health and the relationship may be mediated by poor health for the individuals with less elastic arteries. However, these relationships were independent of blood pressure (either not related to variable in question or continued to be related to arterial elasticity even after adjusting for blood pressure) or blood lipids (none of the blood lipids even approached a significant relationship with any performance measure). Another explanation for the aforementioned findings may be that increased arterial elasticity improves the steady state flow to the capillaries. Consistent with this premise lowering of oxygen tension in the brain can amplify central fatigue [21].

We found differences between the voluntary and electrically-elicited muscle fatigue. During voluntary contractions the neuromuscular system utilizes multiple strategies to offset fatigue. For example, recruiting motor units in an orderly manner such that fatigue resistant muscle is recruited first or altering activated motor units to maintain force production is a common way in which force production is preserved. However, during electrical stimulation, these strategies are not available because motor units are recruited in a nonselective, spatially fixed, and temporally synchronous pattern [17]. We utilized maximum voluntary contractions during our voluntary fatigue protocol to reduce the ability of the neuromuscular system to offset fatigue and be similar to that which is seen during electrically elicited contractions. However, subjects may not have always provided a true “maximum” contraction of the quadriceps, as evidenced from their Central Activation Ratios which were less than 0.95. Because of this, they may have had motor units in reserve that could be called upon when others were fatiguing.

Tiredness was not correlated with measures of voluntary fatigue but was related to the fatigue as a result of the electrical stimulation. The electrical stimulation induced contractions provide information that is specific to the periphery and removes most of the input from the higher central systems. This may provide evidence of a link between peripheral muscle function and central tiredness. The lack of a relationship between the tiredness and voluntary fatigue is probably related to variability in each person's effort at the beginning of the fatigue protocol.

## 5. Conclusions

In conclusion, arterial elasticity is related to strength independent of leg lean tissue (surrogate of leg muscle) and to fatigue independent of ethnic origin, strength, and aerobic fitness. This is especially apparent in the central nervous system where arterial elasticity is independently related to perceptions of fatigue at rest and central fatigue during 30 maximal contractions. These results suggest that arterial health may be involved with the ability for the central nervous system to activate muscle in older women.

## Figures and Tables

**Table 1:** Subject characteristics and physiologic, Fatigue, and performance measures. Sample size is 91 (15 AA and 76 EA) unless otherwise indicated. (mean ± SD).

Age (years)	65.0 ± 3.9
Height (cm)	165.0 ± 5.8
Weight (kg)	73.9 ± 11.4
% Fat	42.8 ± 6.0
Leg lean tissue (kg)	12.9 ± 1.9
VO_2_ max (mL/kg/min, *N* = 77)	23.7 ± 4.1
Treadmill endurance (min, *N* = 77)	13.1 ± 3.0
Loss of energy^a^ (*N* = 74)	0.68 ± 0.68
Fatigue/tiredness^b^ (*N* = 74)	0.53 ± 0.53
Maximum voluntary contraction (N.M, *N* = 40)	116.9 ± 26.7
Maximum voluntary contraction + electrical stimulation (N.M, *N* = 40)	123.6 ± 26.5
VOL (*N* = 40)	22.2 ± 18.6
ESTIM (*N* = 40)	40.2 ± 14.5
CAR1 (*N* = 40)	90.9 ± 6.4
CAR30 (*N* = 40)	89.5 ± 9.1
Large artery elasticity	13.1 ± 4.7
Small artery elasticity	4.0 ± 1.8
SBP (mm Hg, *N* = 90)	126.7 ± 14.9
DBP (mm Hg, *N* = 90)	70.7 ± 10.2

^a^Scored as 4, very low energy, to 0, full of energy; ^b^scored as 4, exceedingly fatigued or tired, to 0, no feelings of fatigue or tiredness.

VOL = % drop in force for 30 voluntary maximal contractions.

ESTIM = % drop in force for voluntary contraction + electrical stimulation during 30 voluntary maximal contractions. CAR1 = Central Activation Ratio for the first repetition in the fatigue test.

CAR30 = Central Activation Ratio for the 30th repetition in the fatigue test.

**Table 2:** Pearson product correlation table.

	Age	% Fat	Leg lean tissue	VO_2_ max	Maximum voluntary contraction	Maximum voluntary contraction + electrical stimulation	Loss of energy	Feelings of tiredness	Large artery elasticity	Small artery elasticity	SBP
Treadmill endurance	−0.38 (<0.01) (*N* = 77)	−0.28 (<0.02) (*N* = 77)	0.01 (0.98) (*N* = 77)	0.54 (<0.01) (*N* = 77)	0.09 (0.64) (*N* = 39)	0.11 (0.58) (*N* = 39)	−0.11 (0.45) (*N* = 71)	0.03 (0.82) (*N* = 71)	0.27 (<0.03) (*N* = 68)	0.03 (0.98) (*N* = 68)	−0.20 (0.06) (67)
Maximum voluntary contraction	−0.24 (0.14) (*N* = 40)	−0.01 (0.97) (*N* = 40)	0.57 (<0.01) (*N* = 40)	0.17 (0.37) (*N* = 39)		0.99 (0.01) (*N* = 40)	−0.11 (0.61) (*N* = 37)	−0.23 (0.28) (*N* = 37)	0.32 (<0.05) (*N* = 40)	0.63 (<0.01) (*N* = 40)	−0.23 (0.16) (*N* = 39)
Maximum voluntary contraction + electrical stimulation	−0.26 (0.12) (*N* = 40)	0.01 (0.95) (*N* = 40)	0.57 (<0.01) (*N* = 40)	0.16 (0.41) (*N* = 39)	0.99 (<0.01) (*N* = 40)		−0.11 (0.60) (*N* = 26)	−0.19 (0.37) (*N* = 26)	0.36 (<0.03) (*N* = 40)	0.65 (<0.01) (*N* = 40)	−0.04 (0.84) (*N* = 39)
CAR1	0.09 (0.60) (*N* = 40)	−0.21 (0.22) (*N* = 40)	0.11 (0.51) (*N* = 40)	0.01 (0.99) (*N* = 39)	0.25 (0.14) (*N* = 40)	0.15 (0.39) (*N* = 40)	−0.06 (0.78) (*N* = 26)	−0.12 (0.57) (*N* = 26)	−0.37 (<0.03) (*N* = 40)	0.06 (0.73) (*N* = 40)	−0.08 (0.83) (*N* = 39)
CAR30	0.02 (0.93) (*N* = 40)	0.07 (0.68) (*N* = 40)	0.10 (0.10) (*N* = 40)	0.10 (0.59) (*N* = 39)	0.12 (0.46) (*N* = 40)	0.04 (0.83) (*N* = 40)	−0.09 (0.68) (*N* = 26)	−0.29 (0.15) (*N* = 26)	−0.34 (<0.04) (*N* = 40)	−0.08 (0.63) (*N* = 40)	−0.05 (0.78) (*N* = 39)
Loss of energy	0.20 (0.09) (*N* = 74)	0.26 (<0.03) (*N* = 74)	−0.02 (0.85) (*N* = 74)	−0.21 (0.12) (*N* = 49)	−0.11 (0.61) (*N* = 26)	−0.11 (0.60) (*N* = 26)		0.49 (<0.01) (*N* = 74)	−0.27 (<0.04) (*N* = 74)	−0.16 (0.20) (*N* = 74)	0.04 (0.74) (*N* = 73)
Feelings of tiredness	0.06 (0.60) (*N* = 74)	0.10 (0.42) (*N* = 74)	−0.07 (0.59) (*N* = 74)	−0.04 (0.76) (*N* = 49)	−0.23 (0.28) (*N* = 26)	−0.19 (0.37) (*N* = 26)	0.49 (<0.01) (*N* = 74)		−0.32 (<0.01) (*N* = 74)	−0.26 (<0.04) (*N* = 74)	0.03 (0.81) (*N* = 73)
Large artery elasticity	−0.27 (<0.01) (*N* = 91)	−0.22 (<0.04) (*N* = 91)	0.02 (0.82) (*N* = 91)	0.34 (<0.01) (*N* = 77)	0.32 (<0.05) (*N* = 40)	0.36 (<0.03) (*N* = 40)	−0.27 (<0.04) (*N* = 74)	−0.32 (<0.01) (*N* = 74)		−0.37 (<0.03) (*N* = 91)	−0.017 (0.89) (*N* = 90)
Small artery elasticity	−0.30 (<0.01) (*N* = 91)	0.17 (0.10) (*N* = 91)	0.38 (<0.01) (*N* = 91)	0.01 (0.98) (*N* = 77)	0.63 (<0.01) (*N* = 40)	0.65 (<0.01) (*N* = 40)	−0.16 (0.20) (*N* = 74)	−0.26 (<0.04) (*N* = 74)	0.25 (<0.02) (*N* = 91)		−0.28 (0.02) (*N* = 90)

^a^Scored as 4, very low energy, to 0, full of energy; ^b^scored as 4, exceedingly fatigued or tired, to 0, no feelings of fatigue or tiredness.

CAR1 = Central Activation Ratio for the first repetition in the fatigue test.

CAR30 = Central Activation Ratio for the 30th repetition in the fatigue test.

SBP = systolic blood pressure.

**Table 3:** Linear regression model for estimation of central fatigue (CAR30) from aerobic fitness, knee extension strength, and large artery elasticity (38 subjects).

Central fatigue	Beta	*R* = 0.49	*P* < 0.02
		Partial *R*	*P*
Constant	0.861		<0.01
VO_2_ max	0.006	0.35	<0.04
Knee extension strength	<0.001	0.08	0.64
Large artery elasticity	−0.008	−0.42	<0.01

**Table 4:** Linear regression models for estimation of treadmill endurance. Model 1 from aerobic fitness, knee extension strength, large artery elasticity, and age. Model 2 from aerobic fitness, knee extension strength, large artery elasticity, and % fat (both models, 39 subjects).

	Beta	*R* = 0.66	<0.01
		Partial *R*	*P*
Treadmill model 1 endurance			
Constant	7.184		0.31
VO_2_ max	0.262	0.47	<0.01
Knee extension strength	0.007	0.09	0.62
Large artery elasticity	0.201	0.37	<0.03
age	−0.128	−0.10	0.46

Treadmill model 2 endurance			
VO_2_ max	0.30	0.53	<0.01
Knee extension strength	0.01	0.10	0.58
Large artery elasticity	0.22	0.40	<0.02
% fat	0.06	0.15	0.39

**Table 5:** Linear regression model for estimation of subjective feelings of fatigue from large and small artery elasticity (64 subjects).

Feelings of tiredness	Beta	*R* = 0.38	*P* < 0.01
		Partial *R*	*P*
Constant	1.361		<0.01
Small artery elasticity	−0.086	−0.22	<0.09
Large artery elasticity	−0.043	−0.29	<0.02

**Table 6:** Linear regression model for estimation of knee extension strength from leg lean tissue, small artery elasticity, and age (*n* = 39).

Knee extension strength	Beta	*R* = 0.65	*P* ≤ 0.01
		Partial *R*	*P*
Constant	118.7		0.08
Leg lean tissue (kg)	0.97	0.08	0.65
Small artery elasticity	6.42	0.52	<0.01
age	−1.02	−0.21	0.22

## References

[1] Yu DS, Lee DT, Man NW (2010). Fatigue among older people: a review of the research literature. *International Journal of Nursing Studies*.

[2] Avlund K (2010). Fatigue in older adults: an early indicator of the aging process?. *Aging*.

[3] Iadecola C (2004). Neurovascular regulation in the normal brain and in Alzheimer’s disease. *Nature Reviews Neuroscience*.

[4] Davenport MH, Hogan DB, Eskes GA, Longman RS, Poulin MJ (2012). Cerebrovascular reserve: the link between fitness and cognitive function?. *Exercise and Sport Sciences Reviews*.

[5] Stoquart-ElSankari S, Balédent O, Gondry-Jouet C, Makki M, Godefroy O, Meyer M-E (2007). Aging effects on cerebral blood and cerebrospinal fluid flows. *Journal of Cerebral Blood Flow and Metabolism*.

[6] Tao J, Jin Y-F, Yang Z (2004). Reduced arterial elasticity is associated with endothelial dysfunction in persons of advancing age: comparative study of noninvasive pulse wave analysis and laser Doppler blood flow measurement. *American Journal of Hypertension*.

[7] Fahs CA, Heffernan KS, Ranadive S, Jae SY, Fernhall B (2010). Muscular strength is inversely associated with aortic stiffness in young men. *Medicine and Science in Sports and Exercise*.

[8] Heffernan KS, Chalé A, Hau C (2012). Systemic vascular function is associated with muscular power in older adults. *Journal of Aging Research*.

[9] Seals DR, DeSouza CA, Donato AJ, Tanaka H (2008). Habitual exercise and arterial aging. *Journal of Applied Physiology*.

[10] Fujimoto N, Prasad A, Hastings JL (2010). Cardiovascular effects of 1 year of progressive and vigorous exercise training in previously sedentary individuals older than 65 years of age. *Circulation*.

[11] Gonzales JU (2013). Gait performance in relation to aortic pulse wave velocity, carotid artery elasticity and peripheral perfusion in healthy older adults. *Clinical Physiology and Functional Imaging*.

[12] Brunner EJ, Shipley MJ, Witte DR (2011). Arterial stiffness, physical function, and functional limitation: the whitehall II study. *Hypertension*.

[13] Austin BA, Popovic ZB, Kwon DH (2010). Aortic stiffness independently predicts exercise capacity in hypertrophic cardiomyopathy: a multimodality imaging study. *Heart*.

[14] Lane AD, Wu P-T, Kistler B (2013). Arterial stiffness and walk time in patients with end-stage renal disease. *Kidney Blood Press Res*.

[15] Hunter GR, McCarthy JP, Bamman MM (2004). Effects of resistance training on older adults. *Sports Medicine*.

[16] Gibbons RJ, Balady GJ, Bricker JT (2002). ACC/AHA 2002 guideline update for exercise testing: summary article. A report of the American College of Cardiology/American Heart Association Task Force on Practice Guidelines (Committee to update the 1997 exercise testing guidelines). *Journal of the American College of Cardiology*.

[17] Bickel CS, Slade JM, Warren GL, Dudley GA (2003). Fatigability and variable-frequency train stimulation of human skeletal muscles. *Physical Therapy*.

[18] Mustata S, Chan C, Lai V, Miller JA (2004). Impact of an exercise program on arterial stiffness and insulin resistance in hemodialysis patients. *Journal of the American Society of Nephrology*.

[19] Beck AT, Steer RA, Brown GK Manual for the Beck depression inventory-II.

[20] Kim J, Heshka S, Gallagher D (2004). Intermuscular adipose tissue-free skeletal muscle mass: estimation by dual-energy X-ray absorptiometry in adults. *Journal of Applied Physiology*.

[21] Rasmussen P, Dawson EA, Nybo L, Van Lieshout JJ, Secher NH, Gjedde A (2007). Capillary-oxygenation-level-dependent near-infrared spectrometry in frontal lobe of humans. *Journal of Cerebral Blood Flow and Metabolism*.

